# Review of Psychological Interventions in Oncology: Current Trends and Future Directions

**DOI:** 10.3390/medicina61020279

**Published:** 2025-02-06

**Authors:** Teodora Anghel, Bratu Lavinia Melania, Iuliana Costea, Oana Albai, Amalia Marinca, Codrina Mihaela Levai, Lavinia Maria Hogea

**Affiliations:** 1 Neuroscience Department, “Victor Babes” University of Medicine and Pharmacy, 300041 Timisoara, Romania; anghel.teodora@umft.ro (T.A.); hogea.lavinia@umft.ro (L.M.H.); 2 Neuropsychology and Behavioral Medicine Center, “Victor Babes” University of Medicine and Pharmacy, 300041 Timisoara, Romania; 3 Psychology Department, West Univerity of Timisoara, 300223 Timisoara, Romania; 4 Internal Medicine Department, “Victor Babes” University of Medicine and Pharmacy, 300041 Timisoara, Romania; albai.oana@umft.ro; 5 Center for Studies and Research in Psychology, Faculty of Psychology, “Tibiscus” University, Lascăr Catargiu 4-6, 300559 Timisoara, Romania; amalia_marinca@yahoo.com; 6 Discipline of Medical Communications, Department 2—Microscopic Morphology, “Victor Babeș” University of Medicine and Pharmacy, 300041 Timisoara, Romania; codrinalevai@umft.ro

**Keywords:** psychotherapy, cancer, emotional and spiritual well-being, meaning-centered psychotherapy, mindfulness-based cognitive therapy, supportive–expressive group therapy, cognitive behavioral therapy

## Abstract

*Background and Objectives*: Cancer imposes a profound burden on both physical and psychological health, particularly at advanced stages, which are associated with a poor prognosis and heightened emotional distress. Psychotherapeutic interventions have gained recognition for their role in alleviating distress, enhancing the quality of life, and supporting a holistic approach to cancer care. This review examines the effectiveness of psychotherapeutic interventions in improving psychological well-being in cancer patients. *Materials and Methods*: Conducted as a literature review following PRISMA guidelines, this study analyzed experimental research on psychotherapeutic interventions for cancer patients published in the past decade. Literature searches were performed in PubMed, Google Scholar, the Cochrane Library, Web of Science, PsycINFO, and Consensus, supplemented by manual reference checks. The inclusion criteria focused on randomized controlled trials (RCTs). *Results*: The analysis included 20 RCTs spanning over three decades, evaluating interventions such as individual meaning-centered psychotherapy (IMCP), Mindfulness-Based Cognitive Therapy (MBCT), and supportive–expressive group therapy (SEGT). IMCP emerged as being particularly effective in reducing depression, anxiety, and demoralization while enhancing spiritual well-being. MBCT demonstrated significant reductions in the fear of recurrence, while SEGT effectively addressed traumatic stress and fostered social support. Although the survival benefits were inconsistently reported, psychosocial improvements, including an enhanced quality of life and emotional resilience, were consistently observed. The methodological quality varied, with nine studies meeting high-quality standards. *Conclusions*: Psychotherapeutic interventions, particularly IMCP and MBCT, play a critical role in oncology by alleviating distress, fostering resilience, and improving the quality of life. Integrating these approaches into routine cancer care can ensure a more holistic treatment framework that prioritizes the emotional and psychological needs of patients.

## 1. Introduction

The global cancer burden is a critical focus of research, with significant concern over its incidence and mortality rates. In 2020, the Global Cancer Observatory reported approximately 19.3 million new cancer cases and 10 million cancer-related deaths worldwide [1,2]. Most patients are still definitely diagnosed at an advanced stage, marked by a poor prognosis and increasing incidence and mortality. This stage brings significant psychological burdens, severely impacting their quality of life and highlighting the need for close attention to their mental health [2,3,4,5].

Cancer poses a profound challenge not only to physical health but also to the psychological and emotional well-being of patients. Alongside advancements in medical treatments, the integration of psychotherapeutic interventions has become increasingly recognized for alleviating psychological distress and improving cancer patients’ overall quality of life (QoL) [6,7,8]. The emotional impact of a cancer diagnosis often manifests as anxiety, depression, the fear of progression, and existential distress, emphasizing the need for comprehensive care approaches that address both physical and mental health [9,10].

Advanced cancer is a stage of the disease where it has either spread to nearby tissues or distant organs (metastases) or is deemed inoperable or challenging to treat with curative intent [11].

Various psychotherapeutic modalities have been explored in oncology settings, each offering unique benefits. Cognitive behavioral therapy (CBT) is widely employed to manage anxiety, depression, and maladaptive coping mechanisms. Mindfulness-based interventions, such as Mindfulness-Based Cognitive Therapy (MBCT), have effectively reduced distress and enhanced emotional regulation [10,12]. Supportive–expressive group therapy has been particularly effective in fostering social support and addressing existential concerns [13,14].

Supportive psychotherapy is a therapeutic approach that emphasizes the provision of emotional support and encouragement to patients, particularly those patients facing advanced cancer. Research indicates that supportive psychotherapy is often utilized as a complementary approach alongside other therapeutic modalities, such as CBT and meaning-centered psychotherapy. For instance, a study by Breitbart et al. highlighted the development of individual meaning-centered psychotherapy (IMCP), which is specifically tailored for patients with advanced cancer. This intervention is grounded in the principles of existential psychology and aims to enhance spiritual well-being and a sense of meaning in life, which are critical for patients facing terminal illness [15].

Psychotherapy interventions not only alleviate emotional distress but also improve functional outcomes, enabling patients to navigate better the challenges posed by their illness. For instance, group therapies focusing on emotional expression and coping strategies have shown benefits in reducing the fear of progression and depressive symptoms [2,6]. Additionally, prehabilitation programs incorporating psychological support before treatment have been linked to improved physical and emotional recovery, highlighting the preventive potential of psychotherapeutic care [7,9,16,17,18].

The current understanding in psycho-oncology highlights the critical need to incorporate psychosocial care into standard oncology practices, given its proven benefits in reducing distress, improving the quality of life, and potentially increasing survival rates [19].

Studies have shown that cancer patients often face various psychological challenges, including anxiety, depression, and post-traumatic stress disorder. For example, research on bladder cancer patients found that a substantial number experienced symptoms of depression (77.5%) and anxiety (69.3%) [20]. These findings emphasize the importance of routine psychological assessments and the integration of psychosocial support into cancer treatment plans. However, despite the clear need for such interventions, many healthcare providers fail to address these issues adequately, often due to insufficient training or limited resources [21].

Over time, numerous reviews have significantly contributed to the field of psycho-oncology, with bibliometric studies and reviews highlighting evolving trends and persistent gaps in the research. A bibliometric analysis by Zhang et al. (2020) revealed a global rise in psycho-oncology research from 1999 to 2019, showcasing the diversification of topics such as positive coping mechanisms and their impact on the quality of life for patients and caregivers [22]. Similarly, Ahmad et al. (2022) mapped key themes emerging from 1980 to 2021, including neoplasm, social support, and psychological interventions, affirming psycho-oncology’s growing prominence in comprehensive cancer care [23]. Furthermore, evidence underscores the tangible benefits of integrating psychosocial interventions into cancer care. Kalter et al. (2018) demonstrated through a meta-analysis that such interventions significantly enhance the quality of life, emotional functioning, and social outcomes for cancer patients, reinforcing the necessity of embedding psycho-oncological care within standard treatment protocols [24].

Despite the documented benefits, debates persist regarding the impact of psychotherapy on cancer survival. While some studies suggest potential links between psychosocial interventions and extended survival, others argue that the primary value lies in the improved QoL rather than life extension [14,25]. This ongoing discourse highlights the importance of prioritizing mental healthcare as a core component of cancer management, irrespective of survival outcomes [13,26].

This study aimed to evaluate the effectiveness of psychotherapeutic interventions in improving psychological well-being and the quality of life in cancer patients while identifying the most impactful therapeutic modalities across diverse oncological contexts. A review of various modalities, including cognitive behavioral therapy, mindfulness-based approaches, and group therapies, highlights their contributions to holistic cancer management.

The present article proposes to provide a comprehensive overview of the application of psychotherapeutic interventions in oncology, addressing their relevance and effectiveness across various contexts without limiting the scope to a specific cancer subtype or therapeutic approach. This approach emphasizes their adaptability and applicability to the diverse psychological needs of cancer patients while bridging the gap between niche, cancer-specific studies and generalizable insights that can inform holistic psycho-oncological care.

## 2. Materials and Methods

### 2.1. Objectives and Search Strategy

This paper presents a literature review focused on experimental research examining the effectiveness of psychotherapeutic interventions for cancer patients. The aim was to identify and analyze studies from the past decade, adhering to the guidelines outlined by the Preferred Reporting Items for Systematic Reviews and Meta-Analyses (PRISMA) protocols [27].

The search for articles was conducted using PubMed, Google Scholar, the Cochrane Library, Web of Science, PsycINFO, and the AI tool Consensus with the deadline for searching being December 2024, utilizing the following primary search expression: “Psychotherapy” [MeSH Terms] OR “Psychotherapies” OR “Psychological interventions” OR “Spiritual therapies” OR “Psychotherapy, group” OR “Group psychotherapy” OR “Therapy, group” OR “Group therapy” OR “Supportive-expressive group” AND “Neoplasms” [MeSH Terms] OR “Neoplasia” OR “Neoplasm” OR “Tumor” OR “Cancer” OR “Malignancy” OR “Neoplasm, malignant” OR “CBT” OR “cognitive behavioral therapy” OR “mindfulness” OR “supportive-expressive therapy”. The searches were limited to the title/abstract to capture the most relevant studies. The searches were limited by applying filters for the language (English studies), period (2010–2024), and study type (randomized controlled trials).

The literature search utilized electronic databases to identify articles relevant to the study, complemented by a manual review of references from selected publications. The authors screened titles and abstracts to determine their relevance according to the predefined inclusion criteria.

### 2.2. Inclusion and Exclusion Criteria

Inclusion criteria: (1) patients with advanced cancer, defined as those studies explicitly labeled “advanced cancer” or reporting on patients with metastatic cancer or stages III and IV disease; (2) intervention groups receiving psychotherapy compared to control groups receiving usual care, massage, or supportive psychotherapy; (3) randomized controlled trials; and (4) studies published in English.

Exclusion criteria: (1) animal or in vitro experiments; (2) duplicate studies or those unrelated to the research topic; (3) studies lacking the full text, with only abstracts or unavailable data; (4) conference reports, case reports, meta-analyses, reviews, editorials, letters, protocols, errata, or notes; and (5) studies that included patients suffering from cognitive disorders, particularly those related to orientation or memory.

Psychotherapy interventions included group psychosocial support, cognitive behavioral therapy, individual meaning-centered psychotherapy, positive affect skill interventions, dignity therapy, forgiveness therapy, mindfulness interventions, and focused narrative interventions.

### 2.3. Data Extraction and Evaluation of Quality of Studies

When extracting information from the studies, we concentrated on key elements to ensure a thorough analysis. These elements included the first author’s name and publication year, which helped us effectively organize and cite the studies, as well as the country and study design, which provided insight into geographical and methodological differences. We also collected data on participant groups and sample sizes to assess the statistical power and generalizability of each study. Furthermore, we gathered detailed information about the interventions, including their type, duration, frequency, significant results, and psychosocial or survival outcomes, and noted any limitations or comments. Additional data collected included participants’ demographic characteristics (age and sex) and the type of cancer studied. These specific elements were selected to facilitate meaningful comparisons across studies and to ensure our analysis comprehensively addressed all critical aspects of the research.

The articles included in our research were evaluated for quality using the JBI Checklist for Randomized Controlled Trials [28].

## 3. Results

**Figure 1:**
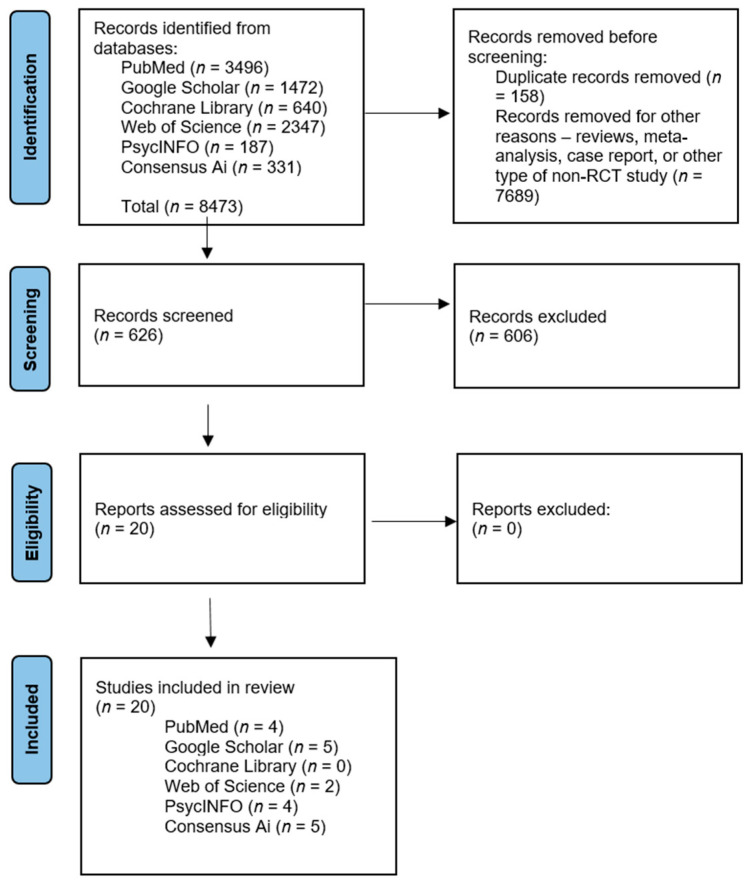
PRISMA flowchart of selected papers for study.

A total of 8473 papers were identified through database searches using PubMed, Google Scholar, the Cochrane Library, Web of Science, PsycINFO, and the AI tool Consensus. The authors eliminated 158 duplicated articles, and 7689 were excluded because they were not aligned with the inclusion criteria (reviews, a meta-analysis, a case report, or another type of non-RCT study). Six hundred and twenty-six papers were selected for the second screening by studying the abstracts. We removed articles that treated subjects with other pathologies, medication, or chemotherapeutic approaches compared with psychotherapy. Twenty key articles on randomized controlled trials examining the impact of psychotherapy on cancer patients were selected (Figure 1). The PRISMA framework guided the selection process, ensuring the systematic and rigorous identification of the most relevant and high-quality studies for analysis.

The articles included in this study exhibited several vital characteristics: the year of publication, sample size, type of cancer addressed, psychotherapeutic approach and intervention details, target condition, outcome measures, research setting, primary findings, and specifics of the treatment program (Table 1).

All studies reviewed (20) were randomized controlled trials (RCTs), ensuring methodological rigor. This design strengthens the reliability of findings on psychotherapeutic interventions for improving cancer patients’ psychological and emotional well-being. The reviewed articles spanned from 1985 to 2020, reflecting over three decades of research on psychotherapeutic interventions for cancer patients. This timeline highlights the evolving focus on addressing emotional, psychological, and existential distress in diverse cancer populations using innovative therapies tailored to improve the quality of life and psychosocial outcomes.

The reviewed studies included a diverse range of participants, totaling 2657 individuals, with 1670 females (63%) and 987 males (37%). Most studies focused on female-dominated cancers like breast cancer, resulting in a higher female representation. Advanced-stage cancer studies showed more of a gender balance. The studies included a minimum of 32 and a maximum of 321 participants, with a median of 125 and an average of 142 participants, reflecting varied sample sizes across the research.

The studies encompassed diverse cancer types, with nine studies focusing on advanced-stage cancers, including works by Fraguell-Hernando C. (2020) [29] and Breitbart W. (2018) [3]. Early-stage breast cancer was examined in one study by Al-Sulaiman R.J. (2018) [30], while breast cancer was the focus of two studies by Andersen B. (2008) [31] and Blanco C. (2018) [32]. Non-metastatic breast cancer appeared in two studies (Marchioro G. 1996 [33]; Park S. 2020 [34]), and metastatic breast cancer in three studies, including a study by Spiegel D. (1989) [13]. Gastrointestinal cancer was explored in one study by Kuchler T. (2007) [35], and mixed cancer types were examined by Greer S. (1992) [36].

The articles were classified based on the psychotherapeutic interventions they employed. Individual meaning-centered psychotherapy (IMCP) was the most frequently studied, with three articles [3,15,29]. Two articles focused on Meaning-Centered Group Psychotherapy (MCGP) [37,38] and supportive–expressive group therapy (SEGT) [39,40].

Single studies explored other therapies, including crisis counseling and psychoeducation [30], biobehavioral group interventions [31], and Mindfulness-Based Cognitive Therapy (MBCT) [34]. Managing Cancer and Living Meaningfully (CALM) was investigated twice [41,42]. Cognitive-based approaches, including Adjuvant Psychological Therapy [36] and Cognitive Psychotherapy with Family Counseling [33], featured prominently.

Relaxation-based interventions, such as Progressive Muscle Relaxation and Guided Imagery (PMR and GI) [43] and clinical hypnosis [44], were explored for their psychological benefits. Additional approaches, such as group therapy alone or with hypnosis [45,46], highlighted diverse applications across cancer care.

The reviewed studies demonstrated significant results across various psychotherapeutic interventions. Individual meaning-centered psychotherapy (IMCP) consistently improved spiritual well-being and the quality of life and reduced demoralization, depression, and anxiety. Meaning-Centered Group Psychotherapy (MCGP) showed similar benefits, with additional reductions in hopelessness, despair, and a desire for hastened death.

Supportive–expressive group therapy (SEGT) significantly reduced traumatic stress symptoms, mood disturbances, and pain perception. Biobehavioral interventions and mindfulness-based therapies, such as MBCT and CALM, effectively reduced psychological distress, attachment anxiety, and the fear of cancer recurrence while enhancing spiritual well-being.

Cognitive and relaxation-based interventions, including Adjuvant Psychological Therapy, PMR and GI, and clinical hypnosis, were effective in reducing depression and improving coping mechanisms, though anxiety outcomes were inconsistent. Group therapies fostered emotional support, better communication skills, and psychological adjustment.

The survival outcomes varied; interventions like IMCP and MCGP did not demonstrate survival benefits, whereas Kuchler’s supportive therapy and Spiegel’s group therapy reported improved survival rates. Across all studies, enhanced emotional well-being, reduced psychological distress, and an improved quality of life were the expected outcomes, underscoring the psychosocial benefits of these interventions in cancer care.

This classification reflects the wide range of psychotherapeutic strategies studied for improving psychological outcomes in cancer patients.

High-quality studies (9 articles) demonstrated robust methodologies and significant findings, while medium-quality (10 articles) studies faced limitations. The sole low-quality study had methodological weaknesses and limited generalizability.

**Table 1:** Randomized control trial studies of psychotherapy among cancer patients.

Study (Year)	Number of Participants	Type of Cancer	Intervention	Duration	Significant Results	Psychosocial/Survival Outcome	Limitations/Comments	Quality (JBI Checklist)
Fraguell-Hernando C. (2020) [29]	*n* = 32 (16 female, 16 male)	Advanced-stage cancer	Individual Meaning-Centered Psychotherapy Palliative Care (IMCP-PC) vs. counseling	Three sessions of 45–60 min each, conducted over four weeks.	The IMCP-PC group showed significant improvements in reducing demoralization, anxiety, depression, and emotional distress compared to the control group. Counseling only showed a significant reduction in demoralization.	Enhanced emotional well-being and reduced psychological distress, anxiety, and depression in the IMCP-PC group, with no significant differences in survival noted.	The sample size was small and the dropout rate was high due to the advanced disease stage.	Medium
Al-Sulaiman R.J (2018) [30]	*n* = 201(all female)	Early-stage breast cancer	Crisis counseling and psychoeducation	Six sessions, each lasting 60–90 min, conducted over 12 weeks.	Both interventions improved psychological well-being and quality of life.	Improved psychological well-being and quality of life in the short and long term; no significant changes in other survival outcomes were reported.	The study was limited by a lack of placebo interventions for the control group, the absence of mid-term evaluations, and variability in the delivery of interventions.	Medium
Andersen B. (2008) [31]	*n* = 227 (all female)	Breast cancer	A biobehavioral group intervention	A total of 26 sessions (39 h of therapy) delivered over 12 months.	The intervention group showed a significantly reduced risk of breast cancer recurrence, death from breast cancer, and death from all causes.	Enhanced psychological well-being and significant survival benefits, including reduced recurrence and mortality rates.	The study focused exclusively on women with breast cancer, limiting generalizability to other cancers or male patients.	High
Blanco C. (2018) [32]	*n* = 134 (all female)	Breast cancer	Comparison of three evidence-based therapies: Interpersonal Psychotherapy (IPT), Problem-Solving Therapy (PST), and Brief Supportive Psychotherapy (BSP)	Weekly sessions for 12 weeks (45 min).	All three interventions significantly improved depressive symptoms and quality of life, with comparable effect sizes.	Enhanced psychological well-being and reduced depressive symptoms across all interventions—no significant differences between therapy types.	High dropout rates (37–52%) and lack of between-group outcome differences limit generalizability.	Medium
Breitbart W. (2010) [37]	*n =* 90 (44 male, 46 female)	Advanced-stage cancer	Meaning-Centered Group Psychotherapy (MCGP) compared with Supportive Group Psychotherapy (SGP)	Eight weekly sessions, each lasting 90 min.	MCGP demonstrated significantly more significant improvements in spiritual well-being and a sense of meaning compared to SGP.	Enhanced spiritual well-being and meaning were observed, contributing to an improved quality of life at the end of life.	Due to the participants’ advanced disease stages, the study experienced high attrition rates. The small sample size limits the statistical power.	Medium
Breitbart W. (2012) [15]	*n* = 120 (72 female, 48 male)	Advanced-stage cancer	IMCP compared with therapeutic massage (TM) as a control	Seven weekly sessions, each lasting 1 h.	IMCP demonstrated more significant improvements in spiritual well-being, sense of meaning, and quality of life than TM.	Enhanced well-being and quality of life, with short-term improvements in psychological and symptom-related distress.	High attrition rate, small sample size for follow-up analysis, and lack of no-treatment control group.	Medium
Breitbart W. (2015) [38]	*n* = 253 (176 female, 77 male)	Advanced-stage cancer	MCGP compared to Supportive Group Psychotherapy (SGP)	Eight sessions, each lasting 90 min.	MCGP significantly improved spiritual well-being and quality of life and reduced depression, hopelessness, and physical symptom distress compared to SGP.	Enhanced spiritual well-being and existential quality of life, reduced psychological distress and despair-related outcomes.	Challenges included high attrition rates (32%), the absence of a distress threshold for inclusion, and a lack of mid-treatment assessments.	High
Breitbart W. (2018) [3]	*n* = 321 (230 female, 91 male)	Advanced-stage cancer	IMCP compared to supportive psychotherapy (SP) and Enhanced Usual Care (EUC).	Seven weekly sessions, each lasting 1 h.	IMCP significantly improved the quality of life, spiritual well-being, and sense of meaning while also reducing anxiety. The effects were minor to moderate compared to EUC.	Enhanced spiritual well-being and quality of life were observed.	The high attrition rate (EUC group) and overrepresentation of women in the sample.	High
Goldberg R.J. (1985) [45]	*n* = 36	Advanced-stage cancer	Group therapy intervention combining supportive and expressive components	Weekly sessions over three months.	Participants reported improved psychological well-being, including reduced anxiety and depression and enhanced coping mechanisms. No significant physical health outcomes.	Improved emotional support and coping skills, with better perceived quality of life; no clear survival benefits were reported.	The small sample size, lack of a control group, and limited diversity reduce the generalizability of the findings.	Low
Greer S. (1992) [36]	*n* = 174 (124 female, 32 male)	Mixed cancer types	Adjuvant Psychological Therapy is a brief, problem-focused cognitive behavioral treatment program	Approximately 6 sessions, each lasting at least one hour, were conducted over eight weeks.	Therapy significantly reduced anxiety, depression, helplessness, and fatalism. Improvements in psychological distress and coping mechanisms.	Enhanced emotional well-being and reduced psychological morbidity, with improved quality of life indicators.	High attrition rate; therapy delivery inconsistencies due to patient circumstances.	Medium
Kuchler T. (2007) [35]	*n* = 271 (132 female, 139 male)	Gastrointestinal cancer	Individualized psychotherapeutic support during the hospital stay, incorporating supportive therapy, crisis intervention, relaxation training, and cognitive–existential approaches	A median of 6 sessions, lasting approximately 222 min in total over the hospital stay.	Patients receiving psychotherapeutic support demonstrated significantly improved 10-year survival rates compared to the control group.	Enhanced coping, reduced emotional distress, and an increased “fighting spirit”, which contributed to long-term survival benefits.	The study was limited by its reliance on patient-reported data for some variables and its inclusion of benign tumors.	High
Lo C. (2016) [41]	*n* = 60 (42 female, 18 male)	Advanced metastatic cancer	Managing Cancer and Living Meaningfully (CALM), a brief, individualized psychotherapy	3–6 sessions delivered over 3–6 months, tailored to individual needs.	CALM showed potential benefits in reducing depressive symptoms, attachment anxiety, and attachment avoidance, with a higher likelihood of improvement compared to usual care.	Improvement in depressive symptoms and attachment security. The therapy’s supportive nature and emotional exploration provided notable psychosocial benefits.	Small sample size and contamination of usual care group.	Medium
Marchioro G. (1996) [33]	*n* = 36 (all female)	Non-metastatic breast cancer	Weekly individual cognitive psychotherapy sessions paired with bimonthly family counseling	Weekly individual sessions lasting 50 min for 9 months, with bimonthly family counseling.	The intervention group showed significant improvements in depression and the quality of life.	Enhanced coping mechanisms, improved quality of life, and significant reductions in depressive symptoms.	Small sample size, generalizability is limited due to homogenous participant pool, and potential bias from self-reported measures exists.	Medium
Park S. (2020) [34]	*n* = 74 (all female)	Non-metastatic breast cancer	Mindfulness-Based Cognitive Therapy (MBCT)	Eight weekly group sessions, each lasting two hours, with daily homework assignments (20–45 min).	MBCT significantly reduced psychological distress, fatigue, and fear of cancer recurrence. It also improved spiritual well-being, quality of life, and mindfulness skills.	Enhanced psychological and spiritual well-being, improved quality of life, and reduced fear of cancer recurrence, with no direct survival outcomes reported.	The study was conducted in a single facility. The sample size was relatively small, and longer term effects were not evaluated.	High
Rodin G. (2018) [42]	*n* = 305 (182 female, 123 male)	Advanced-stage cancers	CALM, a brief, manualized, supportive–expressive psychotherapeutic intervention	Three to six individual therapy sessions delivered over 3 to 6 months, each lasting 45–60 min.	CALM significantly reduced depressive symptoms and increased end-of-life preparedness compared to usual care. Improvements in depressive symptoms were clinically meaningful and more pronounced at 6 months.	Enhanced emotional and spiritual well-being and better preparation for end-of-life concerns, but there were no direct survival benefits.	The study was conducted at a single site, limiting the generalizability. The attrition was moderate, primarily due to disease progression.	High
Spiegel D. (1989) [46]	*n* = 86 (all female)	Metastatic breast cancer	Weekly supportive group therapy, including self-hypnosis for pain management	Weekly 90 min sessions over one year, alongside standard oncological care.	The intervention group showed significantly improved survival. Participants reported reduced pain, better emotional coping, and enhanced communication skills.	The intervention contributed to improved well-being and a significant increase in the survival time.	The study did not control for all possible confounding variables, and the specific mechanisms linking group therapy to extended survival were unclear.	High
Classen C. (2001) [39]	*n* = 125 (all female)	Metastatic breast cancer	Supportive–expressive group therapy (SEGT)	Weekly 90 min group sessions for one year.	SEGT significantly reduced traumatic stress symptoms (e.g., intrusion and avoidance) compared to the control group.	Enhanced emotional well-being with reduced traumatic stress symptoms and mood disturbances, notably when death-proximal assessments were excluded.	The study did not assess survival outcomes and faced challenges such as group attendance variability.	High
Goodwin P.J (2001) [40]	*n* = 125 (all female)	Metastatic breast cancer	Supportive–expressive group therapy (SEGT)	Weekly 90 min group sessions for one year.	SEGT improved the mood and reduced the perception of pain, particularly in women who were more distressed at baseline.	Improved emotional well-being and reduced pain perception were observed, but no survival benefits.	The study’s results were influenced by high variability in the baseline distress levels. The absence of survival benefits contradicts earlier findings.	High
Liossi C. (2001) [44]	*n* = 50 (23 female, 27 male)	Advanced-stage cancer	Clinical hypnosis as an adjunct to standard medical and psychological care	Four weekly sessions, each lasting 30 min, combined with ongoing standard care.	The hypnosis group showed significant reductions in anxiety and depression compared to the standard care group. Participants in the hypnosis group also reported an improved overall quality of life, with better psychological adjustment and coping mechanisms.	Enhanced emotional well-being and quality of life, with improvements in psychological distress and coping; no specific survival outcomes were reported.	The study was limited by its small sample size, which included only terminally ill patients who were well enough to participate, and the absence of a placebo control group.	Medium
Sloman R. (2002) [43]	*n* = 56 (26 female, 30 male)	Advanced-stage cancer	Progressive Muscle Relaxation (PMR) and Guided Imagery (GI) were assessed individually and in combination	Three weeks, with twice-daily practice at home and bi-weekly supervised sessions lasting approximately 30 min each.	PMR, GI, and a combination significantly improved depression and the quality of life compared to the control group. However, none of the treatments reduced anxiety levels.	Enhanced quality of life and reduced depression, but anxiety remained unaffected.	The small sample size limited the statistical power, and the Hospital Anxiety and Depression (HAD) scale may not have been sensitive enough to detect small changes in anxiety.	Medium

*n* = number of patients included in each study.

## 4. Discussion

Cancer has been considered, since the 20th century, a disease that endangers patients’ lives, both through its associated symptoms and its impact on their well-being and quality of life. Consequently, psychotherapy has been regarded as an essential component of comprehensive care for cancer patients. In 1983, Derogatis et al. found that over 50% of cancer patients are diagnosed with at least one psychiatric disorder. The most common diagnosis was depressive disorder, including adjustment disorder with depressed mood (12%), mixed emotional features (13%), unipolar major depression (4%), or anxiety [47]. Also, Spiegel et al., in 1989, suggested that psychotherapy might improve survival rates in cancer patients, although subsequent research has produced mixed results [13].

When looking for the articles in our study, we identified diverse intervention strategies in cancer care. Addressing the challenges in the lives of patients with cancer, we discovered that many strategies have been applied, such as IMCP, which has been extensively studied, underscoring its prominence and effectiveness in improving patients’ spiritual well-being, quality of life, and psychological resilience. Its individualized approach provides patients with a structured way to find meaning and purpose despite their diagnosis, which is crucial for managing existential distress. This therapeutic method is particularly valuable for patients in advanced stages of cancer, where maintaining their quality of life is a primary focus [3]. Other therapies applied are MCGP or SEGT, which have demonstrated significant benefits, particularly in enhancing social connectedness, spiritual well-being, and coping mechanisms. These group-based approaches offer additional advantages by fostering community and shared experiences among patients. MCGP’s structured focus on existential themes and SEGT’s emphasis on emotional expression and support provide complementary benefits that address the multifaceted nature of psychological distress in cancer care [38].

Crisis counseling and psychoeducation and biobehavioral group interventions focus on immediate psychological support and coping strategies, making them especially useful for early-stage cancer patients. Psychoeducation empowers patients with knowledge and tools to manage their psychological and emotional responses, enhancing their self-efficacy and mental health [48]. Similarly, MBCT promotes emotional regulation and reduces distress by encouraging mindfulness and present-moment awareness. Its utility extends to alleviating anxiety, depression, and the fear of recurrence, contributing to improved psychological and spiritual well-being [34]. The diversity of mindfulness interventions, including group-based programs and digital applications, allows for tailored approaches to meet the unique needs of cancer patients. For example, a randomized controlled trial exploring a smartphone app-based mindfulness intervention found it feasible and beneficial for cancer survivors to use, addressing their specific psychosocial needs [49]. Additionally, integrating mindfulness with cognitive behavioral strategies has shown promise in enhancing psychological flexibility, which is crucial for coping with the uncertainties associated with cancer [50,51]. This combination of approaches targets immediate psychological symptoms and fosters long-term resilience and well-being.

The CALM intervention has gained traction for its integrative approach, addressing emotional, existential, and practical concerns. It provides a structured framework for patients to navigate their cancer journey, prepare for end-of-life considerations, and strengthen emotional bonds with loved ones. CALM has shown particular promise in reducing depressive symptoms and improving attachment security, making it a vital intervention for advanced cancer care [41,42].

Cognitive-based therapies, such as Adjuvant Psychological Therapy and Cognitive Psychotherapy with Family Counseling, further highlight the importance of integrating structured cognitive behavioral approaches with family dynamics. These therapies address both individual psychological distress and relational challenges, fostering a supportive environment for emotional recovery [52].

The papers included in this study have demonstrated varying degrees of effectiveness in alleviating psychological distress, with interventions such as IMCP, MCGP, SEGT, and others yielding promising results across multiple dimensions.

IMCP consistently demonstrated reductions in anxiety and emotional distress among patients with advanced-stage cancer. For example, Fraguell-Hernando et al. (2020) showed significant improvements in anxiety through just three sessions, indicating the effectiveness of a structured, meaning-focused approach even in a brief format [29]. Similarly, Breitbart (2012, 2018) confirmed that IMCP led to enhanced spiritual well-being and reduced anxiety compared to controls [3,15]. Group interventions such as MCGP have shown moderate reductions in anxiety, although their primary focus remains on meaning and the existential quality of life [38]. Progressive Muscle Relaxation (PMR) and Guided Imagery (GI) failed to reduce anxiety in advanced cancer patients significantly. This may be due to the intrinsic difficulty of alleviating existential anxiety through relaxation-based techniques alone [43]. IMCP and MCGP were highly effective in reducing depression across studies. Breitbart (2010, 2012) highlighted that meaning-focused therapies substantially lowered depressive symptoms and hopelessness. In MCGP, the structured exploration of meaning and group support also improved outcomes [15,37]. Cognitive behavioral approaches like Adjuvant Psychological Therapy showed lasting reductions in depression, helplessness, and fatalism, demonstrating their utility in bolstering coping mechanisms [36]. Mindfulness-Based Cognitive Therapy significantly reduced depression and fatigue, with improvements in emotional regulation and mindfulness skills, highlighting its potential to address psychological distress holistically [34].

SEGT demonstrated reductions in mood disturbances and traumatic stress symptoms, notably when death-proximal assessments were excluded, showcasing its efficacy in managing long-term emotional distress [39,40]. Psychoeducation combined with crisis counseling yielded significant emotional benefits, including improved psychological well-being and emotional functioning, particularly in early-stage breast cancer patients [30].

While most interventions primarily targeted psychological distress, a few, such as SEGT and IMCP, demonstrated survival benefits. For instance, IMCP and SEGT studies reported an improved quality of life and emotional well-being alongside increased survival in some cases, linking psychological relief to potential physiological advantages [53].

Authors like Malik et al., 2022, and Charalambous et al., 2017, and others suggest that psychotherapeutic interventions tailored to the unique needs of cancer patients are essential in mitigating psychological distress and anxiety and ultimately improving survival outcomes. The psychological burden experienced by cancer patients is profound, often exacerbated by the stress of diagnosis, treatment, and the existential threats posed by the disease itself. A comprehensive understanding of the psychological landscape of cancer patients reveals that interventions such as CBT, psychoeducation, and supportive–expressive therapy can significantly alleviate anxiety and improve the quality of life among these individuals [54,55,56,57].

In our research, gender appeared to play a critical role in cancer-focused psychotherapeutic interventions. Studies exclusively involving women with breast cancer [30,31,32,34] focused on early-stage cases and psychosocial interventions, such as crisis counseling, psychoeducation, biobehavioral therapy, and mindfulness-based therapy. These interventions were effective in improving psychological well-being, reducing depressive symptoms, and enhancing the quality of life, but survival outcomes were not consistently measured or improved. For advanced-stage cancers involving both genders [37,38,43], interventions such as MCGP and PMR were aimed at addressing more severe psychological and existential distress, emphasizing the importance of reducing depression and improving emotional well-being. Studies targeting mixed-gender samples with advanced cancers [42,43,44] highlighted that interventions like CALM therapy and clinical hypnosis were effective in reducing depression and anxiety. However, outcomes like survival or existential quality varied depending on the type of cancer and the stage.

The prominence of breast cancer-focused studies [30,31,32,34] reflects both the high prevalence of this cancer and its impact on psychosocial health. These studies frequently measured outcomes like the fear of recurrence, depressive symptoms, and emotional well-being, suggesting tailored interventions for gender-specific concerns in breast cancer. Psychotherapeutic approaches in early-stage cancer patients often showed more robust psychosocial improvements, as patients had fewer existential concerns and may have been more receptive to interventions focused on coping and emotional adjustment. For advanced-stage cancer patients, interventions such as MCGP and CALM therapy were designed to address existential distress and spiritual well-being. These studies demonstrated improvements in the quality of life and reductions in psychological distress, though attrition rates were often high due to disease progression. Greer’s (1992) study on mixed cancer types underscored the efficacy of brief cognitive behavioral therapy in reducing the psychological morbidity regardless of the cancer type [36]. However, attrition rates and patient-specific barriers sometimes limit broader application.

Gender-specific factors and cancer stages significantly shaped the design and effectiveness of psychotherapeutic interventions [58,59,60]. While early-stage interventions predominantly focused on quality-of-life improvements, advanced-stage therapies addressed deeper existential and spiritual needs, often yielding limited survival benefits but meaningful psychosocial outcomes [61,62]. Tailored approaches for gender- and cancer-specific needs remain crucial for maximizing the impact of these therapies.

The role of psychoeducation cannot be overstated, as it equips patients with the knowledge and coping strategies necessary to manage their condition effectively. Research has shown that patients who receive adequate information about their diagnosis and treatment are better able to cope with the psychological aspects of cancer, leading to lower levels of anxiety and depression [63]. This is particularly important in the context of cancer care, where misinformation or a lack of information can exacerbate feelings of helplessness and anxiety [63,64]. Moreover, the psychological impact of cancer is not uniform; it varies significantly across different demographics, including ages, genders, and types of cancer. For example, younger cancer survivors often report higher levels of psychological distress compared to older adults, highlighting the need for tailored interventions that consider these demographic factors [65,66]. Gender differences in anxiety levels among cancer patients have also been documented, suggesting that interventions should be sensitive to these variations to be effective [67,68].

Analyzing the limitations of the papers included in our study highlighted several challenges in conducting psychotherapeutic interventions for cancer patients, emphasizing the need for methodological rigor and practical adjustments. Many studies faced small sample sizes and high dropout rates, particularly in advanced-stage cancer patients, due to disease progression and logistical difficulties in participation. For example, the study by Fraguell-Hernando et al., 2020 [29], was hindered by high attrition and difficulty recruiting patients in home care settings, reflecting the challenges of working with severely ill populations. Small sample sizes, like those in Sloman et al., 2002, and Liossi et al., 2001, limited the statistical power and generalizability [43,44]. Several studies, including those by Al-Sulaiman et al., 2018 [30], and Breitbart et al., 2015, 2018, had samples overrepresented by specific demographics, such as women with breast cancer or well-educated participants [3,38]. This restricts the applicability of the findings to broader populations, particularly males or patients with different cancers. Andersen et al., 2008, for instance, focused solely on women with breast cancer, which limits insights into other cancer types or mixed-gender contexts [31].

The absence of placebo interventions or active control groups in some studies, such as Al-Sulaiman et al., 2018, and Liossi et al., 2001 [30,44], raises concerns about the reliability of the findings. Additionally, the lack of long-term follow-ups in studies like Park et al., 2020, and Marchioro et al., 1996 [33,34], limits the understanding of the sustained benefits from interventions. Studies like Breitbart et al., 2010 [37], faced difficulty in generalizing results due to their focus on advanced cancer stages. Similarly, studies like Lo et al., 2016 [41], noted contamination in usual care groups, which may have diluted the observed intervention effects.

The findings discussed suggest that depression and anxiety are common and significant issues among patients with advanced cancer and can be effectively alleviated through psychotherapeutic interventions. While the review does not clarify the cost-effectiveness of these therapies, it highlights the challenge of providing long-term, continuous care due to the need for trained mental health professionals. Nonetheless, integrating psychological interventions into routine cancer care appears essential for managing depression in this population. Future research should prioritize assessing the cost-effectiveness and developing scalable, affordable interventions tailored to patients with advanced cancer.

Key questions remain regarding the efficacy of psychotherapy for depression in patients with incurable cancer. Most studies reviewed assessed the impact of interventions during or immediately after the treatment process, leaving the long-term effects uncertain [69,70,71]. Additionally, many participants were not clinically diagnosed with depression, limiting the ability to evaluate the interventions’ effectiveness in addressing clinical depression specifically [72,73]. Addressing these gaps in future research is crucial.

This review has several limitations that should be acknowledged. Many of the included studies had small sample sizes, and only twenty were included in the review. As highlighted in the previous literature, the potential for outcome reporting bias cannot be ruled out [74].

Another limitation lies in the undefined physical status of participants, such as their functional capacity or estimated survival. Since the studies did not include critically terminal patients with survival estimates of only a few months, the findings may not apply to individuals in the final stages of cancer. This gap underscores the need for more targeted research in end-stage cancer populations.

Despite these limitations, the review highlights the significant potential of psychotherapy to alleviate psychiatric symptoms associated with cancer in advanced cancer patients. These findings warrant further exploration, particularly to assess the effectiveness of psychotherapy for clinically diagnosed depression in terminally ill patients. Future studies should address these gaps with larger, more representative samples and focus on refining interventions to meet the unique needs of this population.

Future research must focus on developing cost-effective, scalable, and culturally sensitive models tailored to diverse patient populations to advance this field. By addressing the long-term efficacy and accessibility, psychotherapeutic interventions can become a cornerstone of comprehensive cancer care, offering patients the emotional support they need to navigate their journey with dignity and resilience.

## 5. Conclusions

This review underscores the pivotal role of psychotherapy in enhancing the emotional and psychological well-being of cancer patients. Interventions such as CALM and meaning-centered psychotherapy not only alleviate depression and anxiety but also foster spiritual well-being and strengthen coping mechanisms. Among these, IMCP and MBCT stand out as being particularly effective. IMCP addresses existential concerns and promotes psychological and spiritual resilience, especially for patients in advanced stages. Similarly, MBCT significantly reduces anxiety, depression, and fatigue while improving emotional regulation and mindfulness. While the survival benefits of psychotherapy remain uncertain, some interventions show promise, highlighting the complex interplay between psychological care and physical outcomes. These findings reinforce the necessity of integrating psychotherapeutic approaches into routine oncology care, ensuring a holistic treatment strategy prioritizing the quality of life.

## Data Availability

All data discussed in this review are available in the original publications cited in the reference list.

## References

[1] Sung H., Ferlay J., Siegel R.L., Laversanne M., Soerjomataram I., Jemal A., Bray F. (2021). Global Cancer Statistics 2020: GLOBOCAN Estimates of Incidence and Mortality Worldwide for 36 Cancers in 185 Countries. CA Cancer J. Clin..

[2] Wu Y., Pan J., Lu Y., Chao J., Yu H. (2023). Psychotherapy for Advanced Cancer Patients: A Meta-Analysis of the Quality of Life and Survival Assessments. Palliat. Support. Care.

[3] Breitbart W., Pessin H., Rosenfeld B., Applebaum A.J., Lichtenthal W.G., Li Y., Saracino R.M., Marziliano A.M., Masterson M., Tobias K. (2018). Individual Meaning-centered Psychotherapy for the Treatment of Psychological and Existential Distress: A Randomized Controlled Trial in Patients with Advanced Cancer. Cancer.

[4] Teo I., Vilardaga J.P., Tan Y.P., Winger J., Cheung Y.B., Yang G.M., Finkelstein E.A., Shelby R.A., Kamal A.H., Kimmick G. (2020). A Feasible and Acceptable Multicultural Psychosocial Intervention Targeting Symptom Management in the Context of Advanced Breast Cancer. Psycho-Oncology.

[5] De Mol M., Visser S., Aerts J., Lodder P., Van Walree N., Belderbos H., Den Oudsten B. (2020). The Association of Depressive Symptoms, Personality Traits, and Sociodemographic Factors with Health-Related Quality of Life and Quality of Life in Patients with Advanced-Stage Lung Cancer: An Observational Multi-Center Cohort Study. BMC Cancer.

[6] Faller H., Schuler M., Richard M., Heckl U., Weis J., Küffner R. (2013). Effects of Psycho-Oncologic Interventions on Emotional Distress and Quality of Life in Adult Patients With Cancer: Systematic Review and Meta-Analysis. JCO.

[7] Chen Y., Ahmad M. (2018). Effectiveness of Adjunct Psychotherapy for Cancer Treatment: A Review. Future Oncol..

[8] Silver J.K., Baima J. (2013). Cancer Prehabilitation: An Opportunity to Decrease Treatment-Related Morbidity, Increase Cancer Treatment Options, and Improve Physical and Psychological Health Outcomes. Am. J. Phys. Med. Rehabil..

[9] Herschbach P., Book K., Dinkel A., Berg P., Waadt S., Duran G., Engst-Hastreiter U., Henrich G. (2010). Evaluation of Two Group Therapies to Reduce Fear of Progression in Cancer Patients. Support. Care Cancer.

[10] Iordăchescu D.A., Paica C.I., Vladislav E.O., Gică C., Botezatu R., Panaitescu A.M., Ciobanu A.M., Peltecu G., Gică N. (2021). Psychological Intervention in Breast Cancer Pathology. Rom. J. Med. Pract..

[11] DuBenske L.L., Mayer D.K., Gustafson D.H. (2016). Advanced Cancer. Oncology Informatics.

[12] Foley E., Baillie A., Huxter M., Price M., Sinclair E. (2010). Mindfulness-Based Cognitive Therapy for Individuals Whose Lives Have Been Affected by Cancer: A Randomized Controlled Trial. J. Consult. Clin. Psychol..

[13] Spiegel D. (2002). Effects of Psychotherapy on Cancer Survival. Nat. Rev. Cancer.

[14] Spiegel D. (2014). Minding the Body: Psychotherapy and Cancer Survival. Br. J. Health Psychol..

[15] Breitbart W., Poppito S., Rosenfeld B., Vickers A.J., Li Y., Abbey J., Olden M., Pessin H., Lichtenthal W., Sjoberg D. (2012). Pilot Randomized Controlled Trial of Individual Meaning-Centered Psychotherapy for Patients with Advanced Cancer. JCO.

[16] Edwards A., Hulbert-Williams N., Neal R., The Cochrane Collaboration (2004). Psychological Interventions for Women with Metastatic Breast Cancer. Cochrane Database of Systematic Reviews.

[17] Osborn R.L., Demoncada A.C., Feuerstein M. (2006). Psychosocial Interventions for Depression, Anxiety, and Quality of Life in Cancer Survivors: Meta-Analyses. Int. J. Psychiatry Med..

[18] Sheard T., Maguire P. (1999). The Effect of Psychological Interventions on Anxiety and Depression in Cancer Patients: Results of Two Meta-Analyses. Br. J. Cancer.

[19] Caruso R., Breitbart W. (2020). Mental Health Care in Oncology. Contemporary Perspective on the Psychosocial Burden of Cancer and Evidence-Based Interventions. Epidemiol. Psychiatr. Sci..

[20] Draeger D.L., Sievert K.-D., Hakenberg O.W. (2018). Psychosocial Distress in Bladder Cancer Stratified by Gender, Age, Treatment, and Tumour Stage. Urol. Int..

[21] Deshields T., Kracen A., Nanna S., Kimbro L. (2016). Psychosocial Staffing at National Comprehensive Cancer Network Member Institutions: Data from Leading Cancer Centers: Psychosocial Staffing at NCCN Institutions. Psycho-Oncology.

[22] Zhang C., Hu G., Qiu X., Pan L., Wang C. Global Trends of Researches on Pycho-Oncology during 1999–2019: A 21-Year Bibliometric Study Based on VOSviewer 2020. https://www.researchsquare.com/article/rs-22565/v1.

[23] Ahmad T., Ornos E.D.B., Ahmad S., Al-Wassia R.K., Mushtaque I., Shah S.M., Al-Omari B., Baig M., Tang K. (2022). Global Research Mapping of Psycho-Oncology Between 1980 and 2021: A Bibliometric Analysis. Front. Psychol..

[24] Kalter J., Verdonck-de Leeuw I.M., Sweegers M.G., Aaronson N.K., Jacobsen P.B., Newton R.U., Courneya K.S., Aitken J.F., Armes J., Arving C. (2018). Effects and Moderators of Psychosocial Interventions on Quality of Life, and Emotional and Social Function in Patients with Cancer: An Individual Patient Data Meta-analysis of 22 RCTs. Psycho-Oncology.

[25] Coyne J.C., Stefanek M., Palmer S.C. (2007). Psychotherapy and Survival in Cancer: The Conflict between Hope and Evidence. Psychol. Bull..

[26] Cruess D.G., Antoni M.H., McGregor B.A., Kilbourn K.M., Boyers A.E., Alferi S.M., Carver C.S., Kumar M. (2000). Cognitive-Behavioral Stress Management Reduces Serum Cortisol By Enhancing Benefit Finding Among Women Being Treated for Early Stage Breast Cancer: *Psychosom*. Med..

[27] Moher D. (2009). Preferred Reporting Items for Systematic Reviews and Meta-Analyses: The PRISMA Statement. Ann. Intern. Med..

[28] Barker T.H., Stone J.C., Sears K., Klugar M., Tufanaru C., Leonardi-Bee J., Aromataris E., Munn Z. (2023). The Revised JBI Critical Appraisal Tool for the Assessment of Risk of Bias for Randomized Controlled Trials. JBI Evid. Synth..

[29] Fraguell-Hernando C., Limonero J.T., Gil F. (2020). Psychological Intervention in Patients with Advanced Cancer at Home through Individual Meaning-Centered Psychotherapy-Palliative Care: A Pilot Study. Support. Care Cancer.

[30] Al-Sulaiman R.J., Bener A., Doodson L., Bujassoum Al Bader S., Ghuloum S., Lemaux A., Bugrein H., Alassam R., Karim A. (2018). Exploring the Effectiveness of Crisis Counseling and Psychoeducation in Relation to Improving Mental Well-Being, Quality of Life and Treatment Compliance of Breast Cancer Patients in Qatar. IJWH.

[31] Andersen B.L., Yang H., Farrar W.B., Golden-Kreutz D.M., Emery C.F., Thornton L.M., Young D.C., Carson W.E. (2008). Psychologic Intervention Improves Survival for Breast Cancer Patients: A Randomized Clinical Trial. Cancer.

[32] Blanco C., Markowitz J.C., Hellerstein D.J., Nezu A.M., Wall M., Olfson M., Chen Y., Levenson J., Onishi M., Varona C. (2019). A Randomized Trial of Interpersonal Psychotherapy, Problem Solving Therapy, and Supportive Therapy for Major Depressive Disorder in Women with Breast Cancer. Breast Cancer Res. Treat..

[33] Marchioro G., Azzarello G., Checchin F., Perale M., Segati R., Sampognaro E., Rosetti F., Franchin A., Pappagallo G.L., Vinante O. (1996). The Impact of a Psychological Intervention on Quality of Life in Non-Metastatic Breast Cancer. Eur. J. Cancer.

[34] Park S., Sato Y., Takita Y., Tamura N., Ninomiya A., Kosugi T., Sado M., Nakagawa A., Takahashi M., Hayashida T. (2020). Mindfulness-Based Cognitive Therapy for Psychological Distress, Fear of Cancer Recurrence, Fatigue, Spiritual Well-Being, and Quality of Life in Patients With Breast Cancer—A Randomized Controlled Trial. J. Pain Symptom Manag..

[35] Küchler T., Bestmann B., Rappat S., Henne-Bruns D., Wood-Dauphinee S. (2007). Impact of Psychotherapeutic Support for Patients With Gastrointestinal Cancer Undergoing Surgery: 10-Year Survival Results of a Randomized Trial. JCO.

[36] Greer S., Moorey S., Baruch J.D., Watson M., Robertson B.M., Mason A., Rowden L., Law M.G., Bliss J.M. (1992). Adjuvant Psychological Therapy for Patients with Cancer: A Prospective Randomised Trial. BMJ.

[37] Breitbart W., Rosenfeld B., Gibson C., Pessin H., Poppito S., Nelson C., Tomarken A., Timm A.K., Berg A., Jacobson C. (2010). Meaning-centered Group Psychotherapy for Patients with Advanced Cancer: A Pilot Randomized Controlled Trial. Psycho-Oncology.

[38] Breitbart W., Rosenfeld B., Pessin H., Applebaum A., Kulikowski J., Lichtenthal W.G. (2015). Meaning-Centered Group Psychotherapy: An Effective Intervention for Improving Psychological Well-Being in Patients With Advanced Cancer. JCO.

[39] Classen C., Butler L.D., Koopman C., Miller E., DiMiceli S., Giese-Davis J., Fobair P., Carlson R.W., Kraemer H.C., Spiegel D. (2001). Supportive-Expressive Group Therapy and Distress in Patients With Metastatic Breast Cancer: A Randomized Clinical Intervention Trial. Arch. Gen. Psychiatry.

[40] Goodwin P.J., Leszcz M., Ennis M., Koopmans J., Vincent L., Guther H., Drysdale E., Hundleby M., Chochinov H.M., Navarro M. (2001). The Effect of Group Psychosocial Support on Survival in Metastatic Breast Cancer. N. Engl. J. Med..

[41] Lo C., Hales S., Chiu A., Panday T., Malfitano C., Jung J., Rydall A., Li M., Nissim R., Zimmermann C. (2019). Managing Cancer And Living Meaningfully (CALM): Randomised Feasibility Trial in Patients with Advanced Cancer. BMJ Support. Palliat. Care.

[42] Rodin G., Lo C., Rydall A., Shnall J., Malfitano C., Chiu A., Panday T., Watt S., An E., Nissim R. (2018). Managing Cancer and Living Meaningfully (CALM): A Randomized Controlled Trial of a Psychological Intervention for Patients with Advanced Cancer. J. Clin. Oncol..

[43] Sloman R. (2002). Relaxation and Imagery for Anxiety and Depression Control in Community Patients with Advanced Cancer. Cancer Nurs..

[44] Liossi C., White P. (2001). Efficacy of Clinical Hypnosis in the Enhancement of Quality of Life of Terminally Ill Cancer Patients. Contemp. Hypn..

[45] Goldenberg R., Margaret W. (1985). Psychotherapy for the Spouses of Lung Cancer Patients: Assessment of an Intervention. Psychother. Psychosom..

[46] Spiegel D., Kraemer H.C., Bloom R., Gottheil E. (1989). Effect of psychosocial treatment on survival of patients with metastatic breast cancer. Lancet.

[47] Derogatis L.R. (1983). The Prevalence of Psychiatric Disorders Among Cancer Patients. JAMA.

[48] Edelman S., Craig A., Kidman A.D. (2000). Group Interventions with Cancer Patients: Efficacy of Psychoeducational Versus Supportive Groups. J. Psychosoc. Oncol..

[49] Subnis U.B., Farb N.A., Piedalue K.-A.L., Speca M., Lupichuk S., Tang P.A., Faris P., Thoburn M., Saab B.J., Carlson L.E. (2020). A Smartphone App–Based Mindfulness Intervention for Cancer Survivors: Protocol for a Randomized Controlled Trial. JMIR Res. Protoc..

[50] Zhang Z., Leong Bin Abdullah M.F.I., Shari N.I., Lu P. (2022). Acceptance and Commitment Therapy versus Mindfulness-Based Stress Reduction for Newly Diagnosed Head and Neck Cancer Patients: A Randomized Controlled Trial Assessing Efficacy for Positive Psychology, Depression, Anxiety, and Quality of Life. PLoS ONE.

[51] Carney L.M., Park C.L., Hingorany P. (2023). The Mechanisms of Mindfulness-Based Stress Reduction and Mindfulness-Based Cognitive Therapy for Cancer Patients and Survivors: A Systematic Review. Psychol. Conscious. Theory Res. Pract..

[52] Moorey S., Greer S., Bliss J., Law M. (1998). A Comparison of Adjuvant Psychological Therapy and Supportive Counselling in Patients with Cancer. Psycho-Oncology.

[53] Lai J., Song H., Ren Y., Li S., Xiao F. (2021). Effectiveness of Supportive-Expressive Group Therapy in Women with Breast Cancer: A Systematic Review and Meta-Analysis. Oncol. Res. Treat..

[54] Malik M., Wijaya I.K., Amir N., Putri A.A. (2022). The Effect of Cognitive Behavioral Therapy (CBT) on Reducing the Anxiety Level of Breast Cancer Patients Undergoing Chemotherapy at Hasanuddin University Hospital. JCH.

[55] Charalambous A., Kaite C.P., Charalambous M., Tistsi T., Kouta C. (2017). The Effects on Anxiety and Quality of Life of Breast Cancer Patients Following Completion of the First Cycle of Chemotherapy. SAGE Open Med..

[56] Watts S., Prescott P., Mason J., McLeod N., Lewith G. (2015). Depression and Anxiety in Ovarian Cancer: A Systematic Review and Meta-Analysis of Prevalence Rates. BMJ Open.

[57] Alwhaibi M., AlRuthia Y., Sales I. (2023). The Impact of Depression and Anxiety on Adult Cancer Patients’ Health-Related Quality of Life. JCM.

[58] Vasileva A., Karavaeva T., Lyashkovskaya S. (2017). Typology of Psychotherapeutic Targets and Changesin State of Patients with Neurotic Disorders in Thecourse of Personality-Oriented (Reconstructive)Psychotherapy. Arch. Psychiatry Psychother..

[59] Li C.H., Haider S., Shiah Y.-J., Thai K., Boutros P.C. (2018). Sex Differences in Cancer Driver Genes and Biomarkers. Cancer Res..

[60] Wong E., Bedard G., Pulenzas N., Lechner B., Lam H., Thavarajah N., Holden L., Chow E., Lauzon N. (2013). Gender Differences in Symptoms Experienced by Advanced Cancer Patients: A Literature Review. RHC.

[61] Barth J., Munder T., Gerger H., Nüesch E., Trelle S., Znoj H., Jüni P., Cuijpers P. (2016). Comparative Efficacy of Seven Psychotherapeutic Interventions for Patients with Depression: A Network Meta-Analysis. FOC.

[62] Çevik B.E., Taşkin Yilmaz F., Aldemir K., Yildiz E. (2022). Analysis of cancer patients’ illness acceptance and hope levels as per gender and cancer diagnosis. Int. J. Health Serv. Res. Policy.

[63] Husson O., Mols F., Van De Poll-Franse L.V. (2011). The Relation between Information Provision and Health-Related Quality of Life, Anxiety and Depression among Cancer Survivors: A Systematic Review. Ann. Oncol..

[64] Marino P., Touzani R., Pakradouni J., Ben Soussan P., Gravis G. (2022). The Psychological Distress of Cancer Patients Following the COVID-19 Pandemic First Lockdown: Results from a Large French Survey. Cancers.

[65] Abdelhadi O.A., Pollock B.H., Joseph J.G., Keegan T.H.M. (2022). Psychological Distress and Associated Additional Medical Expenditures in Adolescent and Young Adult Cancer Survivors. Cancer.

[66] Yang Y.-L., Liu L., Wang Y., Wu H., Yang X.-S., Wang J.-N., Wang L. (2013). The Prevalence of Depression and Anxiety among Chinese Adults with Cancer: A Systematic Review and Meta-Analysis. BMC Cancer.

[67] Parás-Bravo P., Paz-Zulueta M., Boixadera-Planas E., Fradejas-Sastre V., Palacios-Ceña D., Fernández-de-las-Peñas C., Alonso-Blanco C. (2020). Cancer Patients and Anxiety: A Gender Perspective. Int. J. Environ. Res. Public Health.

[68] Calys-Tagoe B.N.L., Senaedza N.A.H., Arthur C.A., Clegg-Lamptey J.N. (2022). Anxiety and Depression Among Breast Cancer Patients in A Tertiary Hospital in Ghana. PMJG.

[69] Newell S.A. (2002). Systematic Review of Psychological Therapies for Cancer Patients: Overview and Recommendations for Future Research. CancerSpectrum Knowl. Environ..

[70] Williams S., Dale J. (2006). The Effectiveness of Treatment for Depression/Depressive Symptoms in Adults with Cancer: A Systematic Review. Br. J. Cancer.

[71] Hart S.L., Hoyt M.A., Diefenbach M., Anderson D.R., Kilbourn K.M., Craft L.L., Steel J.L., Cuijpers P., Mohr D.C., Berendsen M. (2012). Meta-Analysis of Efficacy of Interventions for Elevated Depressive Symptoms in Adults Diagnosed with Cancer. JNCI J. Natl. Cancer Inst..

[72] Penrod J.D., Morrison R.S. (2004). Challenges for Palliative Care Research. J. Palliat. Med..

[73] Shelby-James T.M., Hardy J., Agar M., Yates P., Mitchell G., Sanderson C., Luckett T., Abernethy A.P., Currow D.C. (2012). Designing and Conducting Randomized Controlled Trials in Palliative Care: A Summary of Discussions from the 2010 Clinical Research Forum of the Australian Palliative Care Clinical Studies Collaborative. Palliat. Med..

[74] Chan A.-W., Altman D.G. (2005). Epidemiology and Reporting of Randomised Trials Published in PubMed Journals. Lancet.

